# Therapeutic Perspective on Tardive Syndrome with Special Reference to Deep Brain Stimulation

**DOI:** 10.3389/fpsyt.2016.00207

**Published:** 2016-12-26

**Authors:** Ryoma Morigaki, Hideo Mure, Ryuji Kaji, Shinji Nagahiro, Satoshi Goto

**Affiliations:** ^1^Parkinson’s Disease and Dystonia Research Center, Tokushima University Hospital, Tokushima University, Tokushima, Japan; ^2^Department of Neurodegenerative Disorders Research, Graduate School of Medical Sciences, Institute of Biomedical Sciences, Tokushima University, Tokushima, Japan; ^3^Department of Neurosurgery, Graduate School of Medical Sciences, Institute of Biomedical Sciences, Tokushima University, Tokushima, Japan; ^4^Department of Clinical Neuroscience, Graduate School of Medical Sciences, Institute of Biomedical Sciences, Tokushima University, Tokushima, Japan

**Keywords:** deep brain stimulation, globus pallidus internus, antipsychotic agents, abnormal involuntary movements, tardive dyskinesia, tardive syndrome, secondary dystonia, pathophysiology

## Abstract

Tardive syndrome (TDS) is a potentially permanent and irreversible hyperkinetic movement disorder caused by exposure to dopamine receptor blocking agents. Guidelines published by the American Academy of Neurology recommend pharmacological first-line treatment for TDS with clonazepam (level B), ginkgo biloba (level B), amantadine (level C), and tetrabenazine (level C). Recently, a class II study provided level C evidence for use of deep brain stimulation (DBS) of the globus pallidus internus (GPi) in patients with TDS. Although the precise pathogenesis of TDS remains to be elucidated, the beneficial effects of GPi-DBS in patients with TDS suggest that the disease may be a basal ganglia disorder. In addition to recent advances in understanding the pathophysiology of TDS, this article introduces the current use of DBS in the treatment of medically intractable TDS.

## Introduction

The term “tardive” originates from the French “*tardif*,” meaning “late”; tardive syndrome (TDS) refers to delayed onset motor disturbances following treatment with psychotropic medication (1, 2). DSM-5 diagnostic criteria for TDS include a history of more than 3 months cumulative exposure to dopamine receptor blocking agents (DRBAs), except in elderly patients in whom 1 month is adequate (3). They also contain the presence of “mild” or “moderate” abnormal involuntary movements (AIMs) in one or more body areas, and the absence of other conditions that might produce AIMs (4, 5).

Tardive syndrome can manifest heterogeneous features of AIMs that comprise dystonia, chorea, athetosis, akathisia, myoclonus, stereotyped behavior, tremor, and tourettism or tics (6–8). Orofacial dyskinesia is the most common symptom in less severe cases, while generalized hyperkinetic movements with predominance of axial dystonia also occur in severe cases (9). Two-thirds of patients with TDS have cervical involvement (10). As many various types of motor symptoms can emerge, it has been suggested that TDS is a more accurate term for the condition than the traditionally used term “tardive dyskinesia (TDD)” (2, 11). TDD is now used to refer to more specific involuntary movements (e.g., lingual–facial–buccal dyskinesia) which are caused by DRBAs (8, 11).

The causative agents are usually typical or atypical antipsychotic drugs (APDs). Recent reports, however, suggest that TDS could also be caused by a wide variety of psychotropic drugs, such as antidepressants and antiparkinsonian medications (7). Systematic overview and meta-regression analyses of 52 randomized controlled trials conducted by Geddes et al. revealed that there are no differential effects between typical and atypical antipsychotics in causing extrapyramidal side effects (12). Recently, O’Brien et al. reviewed studies that investigated the prevalence or incidence of TDS in elderly patients exposed to APDs from 1957 to 2015. The inclusion criteria of this meta-analysis were prospective studies (*n* > 20), which used validated rating scales and research diagnostic criteria (13). According to this meta-analysis, the estimated prevalence for probable TDS—defined according to the Schooler and Kane Research Diagnostic Criteria where abnormal movements in at least one body part are labeled “moderate” and in two or more body parts are rated “mild” (4) — was higher in patients after being treated with typical APDs for 1 year (23 vs. 7%). In more than 50% of cases, TDS was irreversible even after withdrawal from the responsible neuroleptics (14).

In guidelines proposed by the American Academy of Neurology, clonazepam (level B), ginkgo biloba (level B) and amantadine (level C), and tetrabenazine (level C) are recommended for the treatment of TDS (Table 1) (5). Among them, tetrabenazine is most effective at reducing TDS, but has the risk of inducing depression or Parkinsonism (15, 16). Neuroleptic agents cannot be recommended in this guideline since they may cause TDS and mask its symptoms, instead of treating it (5). However, clozapine is the most acceptable alternative for patients with schizophrenia (6). It has the lowest risk among all APDs that cause TDS by inhibiting dopamine D1 and D2 receptors (6, 17). Although its efficacy in reducing TDS is undetermined due to conflicting class III studies, the currently used APDs treatment should be replaced with clozapine as an alternative therapy for suppressing TDS prior to attempting surgical procedures in deep brain stimulation (DBS) clinical trials (18, 19). As published in our previous report (20), accumulating evidence suggests that patients with TDS could be good candidates for undergoing DBS that targets the globus pallidus internus (GPi). Recently, Pouclet-Courtemanche et al. reported a class II evidence trial indicating that GPi-DBS significantly relieves motor symptoms in patients with medically intractable TDS. In this article, we describe recent understandings of the pathophysiology of TDS, and introduce the current use of GPi-DBS in treatment of the disease (19).

**Table 1:** **Evidence-based medical treatments of tardive syndrome**.

Treatments		No. of class I–III studies	Conclusions	Recommendations
Withdrawal of DRBAs		Class III: 3	Conflicting results. In two class III studies, TDS had worsened, while it was unchanged in another	Level U

Acetazolamide with thiamine		Class III: 1	Dyskinesia (AIMS) was reduced by 46 and 41% in older and younger patients, respectively	Level U

Amantadine		Class II: 1 Class III: 2	Dyskinesia (AIMS) was reduced by 15% in one class II study	Level C (short-time use: 7 weeks)

First-generation antipsychotics^a^	Haloperidol	Class II: 2 Class III: 1	TDS was reduced by 67% for up to 2 weeks but akinetic-rigid syndrome was increased in one class II study	Level U
	
	Thiopropazate	Class III: 1	Oral dyskinesia was reduced by 27% after 4 weeks	Level U

Second-generation antipsychotics^a^	Clozapine	Class III: 2	Conflicting results	Level U
	
	Risperidone	Class II: 2 Class III: 1	TDD was reduced. Risperidone is probably effective	Level U
	
	Olanzapine	Class III: 2	TDS (AIMS) was reduced by 30%. Possibly olanzapine reduces TDD	Level U

Dopamine-depleting agents	Tetrabenazine	Class III: 2	TDS (AIMS) is reduced by 54.2%. Long-term TBZ administration can cause parkinsonism	Level C
	
	Reserpine	Class III: 1	TDS was reduced	Level U
	
	α-methyldopa	Class III: 1	TDS was reduced	Level U

Dopamine agonists: bromocriptine		Class III: 1	No TDS reduction	Level U

Cholinergic drugs	Galantamine	Class II: 1	No TDS reduction. Caused parkinsonism. Might not be effective for TDS treatment	Level C

Biperiden (Akineton) discontinuation		Class III: 1	TDS was reduced, but parkinsonism increased	Level U

Antioxidants	Vitamin E	Class II: 6 Class III: 4	Conflicting results. Three class II studies and one class III study failed to show therapeutic effects. In other class II and III studies, vitamin E reduced TDS	Level U
	
	Melatonin	Class II: 2	Conflicting results. Possibly ineffective at low doses, but more effective at higher doses. Data are conflicting	Level U
	
	Selegiline	Class III: 1	TDS reduction relative to the placebo	Level U
	
	Eicosapentaenoic acid	Class II: 1	No TDS reduction. Possibly ineffective	Level C
	
	Ginkgo biloba extract (EGb-761)	Class I: 1	TDS (AIMS) was reduced compared with placebo (2.13 vs. −0.10). Probably useful for treating TDS patients with schizophrenia	Level B
	
	Vitamin B_6_	Class III: 1	TDS (ESRS) was reduced compared with placebo (mean 68.6 vs. 32.8%)	Level U

	Yi-gan san	Class III: 1	TDS (AIMS) was reduced by 56%	Level U

GABA agonists	Clonazepam	Class I: 1	TDS was reduced by 35%	Level B
	
	Baclofen	Class II: 3	Baclofen with neuroleptic agents reduced TDD in two class II studies, but did not reduce TDD when used alone	Level U

Levetiracetam		Class III: 1	Reduced TDD, but dropout rate exceeded 20%	Level U

Calcium channel blocker: diltiazem		Class I: 1	No TDS reduction; probably does not reduce TDD	Level B

Buspirone		Class III: 1	TDS (AIMS) was reduced	Level U

*AIMS, abnormal involuntary movement scale; ESRS, extrapyramidal symptom rating scale; TDD, tardive dyskinesia; TDS, tardive syndromes*.

*^a^Neuroleptics agents cannot be recommended for TDS treatment because of its potential to cause TDS. This table is referred from the guideline of American Academy of Neurology (5)*.

## Pathophysiology of TDS

### Dopamine Receptor Hypersensitivity

Striatal dopamine receptor supersensitivity has so far been the most plausible explanation for development of TDS. Chronic exposure to DRBAs can induce upregulation of postsynaptic dopamine receptors, particularly of the D2 subclass, in the striatum (21). Notably, medications that act on the presynaptic D2 receptors, such as reserpine and tetrabenazine, do not cause TDS (6). The proposed model of a postsynaptic dopamine hypersensitivity mechanism occurring due to upregulation of the D2 receptors is supported by findings obtained from experimental animal models (22–25) and in a human study using positron emission tomography (PET) (26). In the animal models, sub-chronic treatment with antipsychotics increased vacuous chewing movements (VCM) associated with upregulation of striatal D2 receptors (24). Teo et al. hypothesized that hypersensitivity of D2 receptors could cause maladaptive plasticity in the cortico-striatal transmission, resulting in an inability to normalize the miscoded motor program in patients with TDS (27). This notion might be supported by PET findings in patients with TDS (9). In addition to an increase in regional cerebral blood flow during the rest condition in the prefrontal and anterior cingulate cortex and the cerebellum, Thobois et al. (16) reported an excess of brain activity in the prefrontal and premotor cortical areas during motor execution, which might reflect a loss of motor selectivity leading to generation of abnormal movements (9). Trugman et al. hypothesized that the D2 receptor blockade concomitant with repetitive activation of the D1 receptors could be a fundamental cause of TDS (17). This hypothesis might be consistent with the delayed onset of TDS after exposure to neuroleptics and the persistence of TDS even after withdrawal from them (17). In addition, maladaptive changes in non-dopaminergic neurotransmitter systems, such as those involving opioids (enkephalin and dynorphin), glutamate, and acetylcholine, have also been reported in patients with TDS (28, 29) and in animal models of TDS (30–34).

### Neurotoxicity Induced by Oxidative Stress

More recently, oxidative stress has been suggested as a mechanism for TDS pathogenesis. Neuroleptics can exert direct toxic effects on neurons by inhibiting the complex I of the electron transport chain. They also can increase dopamine turnover through chronic dopamine receptor blockade, thereby generating hydrogen peroxide and free radicals, leading to neurotoxicity (8, 35, 36). In animal studies, antipsychotics increase membrane lipid peroxidation, free radical activity, and glutamate transmission, but decrease antioxidant enzyme activity for glutathione (28, 37–39). Defects in the antioxidant systems might cause development of TDS (40). Several authors suggest that oxidative damage leading to neuronal degeneration may explain the irreversibility of TDS (41, 42). In support of this notion, neuroimaging studies using CT and MRI showed that among patients with schizophrenia, a significant reduction in structural volume of the caudate nucleus was found in patients with TDS when compared to non-TDS patients (43–45). Moreover, variances in the gene encoding manganese superoxide dismutase (*MnSOD*) and the gene for an enzyme that eliminates free radicals have also been found to correlate with presence of TDS symptoms (35, 46–49). Based on these findings, a wide variety of antioxidants has been tested in clinical trials (5). The guidelines of the American Academy of Neurology suggest that ginkgo biloba extract (EGb-761) is probably useful (Level B) in TDS therapy (5). Although data conflictingly support or oppose the use of other antioxidative agents, class I and II studies have shown that TDS could be significantly alleviated by vitamin B6, vitamin E, and melatonin (Table 1) (5, 36).

### Genetic Predisposition

Genetic studies suggest that there is an intrinsic susceptibility to develop AIMs in patients with schizophrenia and that the role of antipsychotics is one of promotion or acceleration of rather than causation of symptoms (45, 50). There is solid evidence for a genetic predisposition to TDS (7). Family studies showed that occurrence of TDS was influenced by polymorphisms in the genes coding for the D2 and D3 receptors (*DRD2* and *DRD3*), catechol-O-methyl-transferase (*COMT*), 5-HT2A receptors (*HTR2A*), manganese super-dismutase (*MnSOD*), and cytochrome P450 (*CYP2D6*) (8, 51). Mutations in genes related to GABAergic pathways (*SLCA11, GABRB2*, and *GABRC3*), *N*-methyl-d-aspartate (*NMDA*) receptor (*GRIN2A*), and oxidative stress related genes (*GSTM1, GSTP1, NQO1*, and *NOS3*) are also suggested to play a role in developing TDS (8, 51). Souza et al. reported that *GSK-3*β polymorphism might be a risk factor for TDS in patients with schizophrenia (52). A single nucleotide polymorphism marker located in the 3′-untranslated regulatory region of the *Nurr77* mRNA is nominally associated with risk and severity of AIMs in TDS patients with schizophrenia (53, 54).

### Animal Models of TDS

Rats, mice, and non-human primates have been commonly used as TDS models, in order to investigate disease pathogenesis and evaluate the efficacy of TDS pharmacotherapy. Since the early 1970s, rats that were exposed to dopamine receptor blocking agents for consecutive weeks manifested different patterns of purposeless, chewing activity, which is termed “vacuous chewing movements”(22–25, 30–34). VCM are also observed in mouse models of TDS (55, 56). The VCM induced by haloperidol was further exacerbated by knocking out *Nur77* (57). Knocking out aquaporin-4, however, abolished VCM that were induced by chronic haloperidol treatment (58). The expression patterns of immediate early genes in the striatum, which were induced by clozapine or haloperidol, have been demonstrated using transgenic dopamine D3 receptor knockout mice (59, 60). Thus, transgenic rodent models are beneficial for addressing drug-induced neural changes. Non-human primate model of TDS appeared as early as the late 1970s. Given the marked interspecies difference in susceptibility of New World monkey species, TDS developed in proportions of 0, 45, and 71% in squirrels (*Saimiri sciureus*), capuchins (*Cebus apella*), and marmosets (*Callithrix jacchus*), respectively (61). In non-human primates, chronic APD exposure, typically of haloperidol, for at least 1 year, was required to model TDS (61). Abnormal stereotypical movements observed in non-human primate models of TDS include various orofacial dyskinetic movements, neck rotation, brief back extension, flexion/extension movements of the toes, and upper limb chorea, which persisted for several months following drug withdrawal (61). Since the latency of onset, individual susceptibility, phenomenological expression, and persistence of TDS is similar to humans, non-human primate models of TDS are best suited to address therapeutic issues (61).

## DBS for TDS

### TDS as a Basal Ganglia Circuit Disorder

Accumulating evidence suggests that TDS might result from abnormal plasticity in the motor circuit that links with the basal ganglia (9, 17, 45). Consistent with this concept, TDS was successfully treated with DBS of the GPi, which is the major basal ganglia output nucleus (see Tables 2 and 3). During GPi-DBS surgery in patients with TDS, microelectrode recordings (MERs) of GPi neurons show abnormal bursts and irregular activities (62, 63). In addition, simultaneous recording on pairs of GPi cells also showed a high degree of discharge synchronization (63). By means of a fast Fourier transform analysis, Nandi et al. reported that local field potentials in the GPi showed significant strength of correlation and coherence with the EMG data of AIMs in a patient with TDS (64). Given the evidence that in patients with TDS, GPi cells fired before onset of AIMs, Magariños-Ascone et al. suggest that the burst and irregular patterns of neuronal discharges might indicate an imperfect code that becomes arranged in a confused order at the cortical level, and that GPi-DBS could disrupt these “noisy signals” and allow the motor program to be gated with ease (63). Evidence that GPi-DBS could influence the brain CBF levels in the primary and associative motor cortices has also been reported (9, 62). It has also been noted that not only the GPi but also the STN and thalamus could be targets for DBS in the treatment of TDS (65, 66). These observations indicate that TDS might be a network disorder involving cortico-thalamo-basal ganglia motor circuitry.

**Table 2:** **Reported cases of GPi-DBS in patients with tardive syndrome**.

Reference	*N*	Age/sex	Disease duration, years	Neuroleptics	Indication	Affected regions/type
Trottenberg et al. (67)	1	70/F	6	FLUS	AD, neurosis	Eye, OBL, Cx, Tr, L/Dy

Nandi et al. (64); Yianni et al. (68)^a^	1	40/M	5	HAL, DPD, CPZ	AD, DD, personality disorder	Tr/Dy

Schrader et al. (69)	1	64/F	7	FLUS	AD, DD	OBL, L/CH

Krause et al. (80)	3	(1) 67/F, (2) 53/M, (3) 47/F	(1) 22, (2) 5, (3) 22	NR	NR	NR

Eltahawy et al. (70)	1	53/F	4	PPZ, CPZ	BD	Cx, Tr, L/Dy, CH, akathisia

Trottenberg et al. (82)	5	(1) 70/F, (2) 66/F, (3) 56/F, (4) 30/M, (5) 59/M	NR	(1) FLUS, (2) FLUS, (3) HAL, (4) BPD, LEV, HAL, (5) HAL	(1) AD, (2) DD, (3) BD, (4) SCZ, (5) DD, psychosis	NR/Dy

Franzini et al. (10)	2	(1) 33/M, (2) 30/M	(1) 5, (2) 3	(1) HAL, PIM, RIS, (2) HAL	(1) SCZ, (2) panic disorder	(1) OBL, Cx, Tr, L/Dy, (2) Cx, Tr, L/Dy

Halbig et al. (81)^a^	2	(1) 66/NR, (2) 56/NR	(1) 4, (2) 11	NR	NR	NR

Cohen et al. (84)	2	(1) 44/M, (2) 50/M	(1) 4, (2) 4	(1) HAL, (2) FPZ	(1) SCZ, (2) PTSD	(1) Cx, Tr, L/Dy, (2) Eye, OBL, Cx, Tr/Dy

Starr et al. (83)^a^	4	(1) 36/NR, (2) 47/NR, (3) 59/NR, (4) 36/NR	(1) 7, (2) 4, (3) 20, (4) 10	NR	NR	(1) L/Dy, (2) Face, Cx, L/Dy, (3) Face, L/Dy, (4) generalized/Dy

Damier et al. (18); Thobois et al. (9)	10	(1) 40/F, (2) 33/F, (3) 69/F, (4) 45/M, (5) 51/M, (6) 43/F, (7) 56/F, (8) 27/F, (9) 26/M, (10) 61/F	(1) 2, (2) 4, (3) 4, (4) 2, (5) 6, (6) 9, (7) 3 (8) 3, (9) 4, (10) 3	Neuroleptics	(1)–(4) (7) (10) DD, (5) (6) (8) SCZ, (9) childhood disintegrative disorder	(1) Tr/Dy, (2) Face, L/Dy, CH, (3) Face, Tr/Dy, CH, (4) Tr, L/Dy, CH, (5) Face, Tr, L/Dy, CH, (6) L, Tr/Dy, CH, (7) L/Dy, (8) L, Tr/Dy, (9) L, Tr/Dy, CH, (10) Face, L/Dy, CH

Egidi et al. (85)^a^	5	NR	NR	NR	NR	NR

Kosel et al. (71)	1	62/F	10	Neuroleptics	DD	OBL, L/CH

Magariños-ascone et al. (63)^a^	1	59/F	4	NR	NR	Tr/Dy

Pretto et al. (72)^a^	1	72/F	NR	Neuroleptics	NR	Face, Cx, OBL, Tr, L/Dy

Sako et al. (20)	6	(1) 48/F, (2) 48/F, (3) 30/M, (4) 47/F, (5) 39/M, (6) 55/M	(1) 2, (2) 6, (3) 2, (4) 3, (5) 2, (6) NR	(1) SUL, (2) TPR, (3) RIS, (4) PPZ, (5) PPZ, (6) HAL	(1) (5) DD, (2) BD, (3) SCZ, (4) panic disorder, (6) neurosis	(1) Eye, OBL, Cx/Dy, (2) Cx, Tr, L/Dy, (3) L, Tr/Dy, (4) Cx, Tr/Dy, (5) Cx, L/Dy (6) NR

Gruber et al. (86)	9	(1) 66/F, (2) 70/F, (3) 56/F, (4) 71/M, (5) 38/M, (6) 76/F, (7) 70/F, (8) 75/F, (9) 47/F	(1) 5, (2) 6, (3) 11, (4) 3, (5) 10, (6) 6, (7) 2, (8) 2, (9) 3	(1) FLU, (2) FLU, (3) HAL, (4) PMZ, (5) FPZ, (6) FPX, (7) FLU, (8) MCP, (9) PZ	(1) (3) (4) (7) (9) DD, (2) AD, (5) SCZ, (6) psychosis, (8) gastritis	NR

Katasakiori et al. (73)^a^	1	40/NR	NR	NR	NR	NR

Kefalopoulou et al. (62)	1	42/M	3	LEV	BD	Eye, OBL, Cx, L/CH, Dy

Capelle et al. (87)	4	(1) 45/F, (2) 76/F, (3) 65/F, (4) 48/F	(1) 4, (2) 11, (3) 7, (4) 5	(1) FLUS, (2) HAL, (3) FLUS, PIM, (4) FLUS	(1) (4) DD, (2) nervousness, (3) DD, neurasthenia	(1) Eye, Cx, Tr, L/Dy, CH, (2) Eye, OBL CH, (3) Eye, OBL, Cx/Dy, CH, (4) OBL, L/CH

Chang et al. (88)	5	(1) 36/M, (2) 47/F, (3) 59/M, (4) 36/F, (5) 28/F	(1) 7, (2) 10, (3) 20, (4) 10, (5) 6	(1) TDZ, (2) TDZ, HAL, (3) TDZ, (4) HAL, (5) RIS	(1) SCZ, (2) (3) DD, (4) (5) BD	Generalized/Dy, CH

Kim et al. (74)	1	31/M	6	Neuroleptics	NR	Focal/Dy

Kovacs et al. (75)	1	18/M	0.6	HAL, RIS	SCZ	Face, Tr, L/Dy, CH

Spindler et al. (76)	1	41/M	3.5	TTX	DD	OBL/Dy

Woo et al. (89)	3	(1) 28/F, (2) 46/F, (3) 49/F	NR	(1) ASP, QUE (2) HAL, LG, (3) Fluanxol depot	(1)–(3) SCZ	(1) Face, Cx, Tr, L/Dy, (2) Cx/Dy, (3) Face, Cx, Tr, L/Dy

Boulogne et al. (77)	1	44/M	15	CPZ, FPX, HAL, CMZ, LXP, ALMZ, RIS, OLZ	BD	Cx, Tr/Dy, CH

Trinh et al. (78)	1	27/F	7	RIS	Developmental delay, behavioral disturbance	Eye, Face, Cx, Tr/Dy

Puri et al. (79)	1	51/F	8	HAL	SCZ	OBL, L/Dy

Shaikh et al. (90)	8	(1) 52/F, (2) 58/F, (3) 52/F, (4) 29/M, (5) 62/F, (6) 47/F, (7) 48/F, (8) 38/F	(1) 9, (2) 4, (3) 5, (4) 9, (5) 1, (6) 4, (7) 7, (8) 4	(1) CPZ, TPZ, (2) ARP, (3) ARP, ZPD, (4) ARP, ZPD, RPD, OLZ, (5) PMZ, (6) MCP, (7) RIS, (8) HAL	NR	(1) NR/Dy, (2) NR/Dy, (3) Eye, OBL, Cx, Tr, L/Dy, (4) OBL, Cx, Tr, L/Dy, (5) OBL, Cx, Tr, L/Dy, (6)–(8) NR/Dy

Pouclet-Courtemanche et al. (19)	19	(1) 40/F, (2) 33/F, (3) 69/F, (4) 45/F, (5) 51/F, (6) 43/F, (7) 56/F, (8) 27/F, (9) 26/F, (10) 61/F, (11) 54/F, (12) 59/F, (13) 69/F, (14) 55/F, (15) 64/F, (16) 55/F, (17) 56/F, (18) 58/F, (19) 64/F	(1) 2.4, (2) 5.7, (3) 11, (4) 2.7, (5) 3.1, (6) 3.7, (7) 3.3, (8) 1.4, (9) 4.2, (10) 10.4, (11) 1.8, (12) 7.4, (13) 10.3, (14) 4.4, (15) 1.5, (16) 2.6, (17) 2.7, (18) 38.2, (19) 2.9	(1) ASP, CMZ, (2) HAL, CMZ, FPX, (3) TDZ, (4) PIM, OLZ, (5) HAL, TDZ, OLZ, (6) PIM, HAL, (7) CMZ, (8) ASP, (9) ASP, RIS, (10) LEV, VER, MCP, (11) CMZ, CPZ, (12) CMZ, (13) LEV, SUL, ALMZ, (14) RIS, OLZ, (15) CMZ, (16) MCP, (17) CMZ, ALMZ, (18) HAL, PMP, CMZ, OLZ, (19) ASP, HAL	(1)–(3), (7), (10)–(12), (14), (15), (17)–(19) DD, (4) Tourette syndrome, DD, (5), (6), (8), (13) psychosis, (9) childhood disintegrative disorder, (16) nausea	NR/Dy, CH

*NR, not reported; *Neuroleptics*: ALMZ, alimemazine; ASP, amisulpiride; ARP, aripipirazole; BPD, benperidol; CPZ, chlorpromazine; CMZ, cyamemazine; DPD, droperidol; FLUS, fluspirilene; FPX, flupentixol; FPZ, fluphenazine; LEV, levomepromazine; LG, largactil; LXP, loxapine; HAL, haloperidol; MCP, metoclopramide; OLZ, olanzapine; PIM, pimozide; PMP, pipamperone; PMZ, promethazine; PPZ, perphenazine; PZ, perazine; QUE, quetiapine; RIS, risperidone; RPD, risperdal; SUL, sulpiride; TDZ, thioridazone; TPZ, trifluoperazine; TTX, thiothixene; TPR, tiapride; VER, veralipride; ZPD, ziprasidone. *Indication*: AD, anxiety disorder; BD, bipolar disorder; DD, depressive disorder; PTSD, post-traumatic stress disorder; SCZ, schizophrenia. *Affected regions and type*: Eye, eyelids (blepharospasm), OBL, orobuccolingual; Cx, cervical; Tr, truncal; L, limb; DT, dystonia; CH, choreiform movements*.

*^a^Reports of patients with tardive syndrome within a larger cohort of dystonia patients*.

**Table 3:** **Detailed information about GPi-DBS in patients with tardive syndrome**.

Reference	Evidence level	Target	Active contacts/electrodes used	Mode	Parameters	% improvement	Follow-up time (M)
Trottenberg et al. (67)	4	PV-GPi	C + 1−/Med 3387	M	3.0 V, 150 Hz, 210 µs	BFMDRS-M 73 AIMS 54	6

Nandi et al. (64); Yianni et al. (68)^a^	4	PV-GPi	0–4 + /Med 3387	B	4.0–7.0 V, 130–180 Hz, 150–240 µs	BFMDRS-M 28 BFMDRS-D 39 AIMS 42	12

Schrader et al. (69)	4	GPi	NR/Med 3387	M	6.5 V, 60 Hz, 60 µs	AIMS 63	5

Krause et al. (80)	4	GPi	Most ventral contact/Med 3387	M	NR, 130–180 Hz, 210 µs	BFMDRS-M (1) NR, (2) −2, (3) −1	(1) lost, (2), (3) at most 36

Eltahawy et al. (70)	4	PV-GPi	R C + 2− L C + 2−3−/Med 3387	M	2.6 V, 40 Hz, 210 µs	BFMDRS-M 60	18

Trottenberg et al. (82)	4	PVM-GPi	C + 1− or 2−/Med 3387	M	2.7 V, 144 Hz, 111 µs (mean)	BFMDRS-M (1) 76, (2) 93, (3) 93, (4) 98, (5) 75 BFMDRS-D (1) 80, (2) 100, (3) 100, (4) 100, (5) 100	6

Franzini et al. (10)	4	PVL-GPi	Most ventral contact/Med 3389	M	1.0 V, 130 Hz, 90 µs	BFMDRS-M (1) 86, (2) 88	12

Halbig et al. (81)^a^	4	PVM-GPi	C + 1− or 2−/Med 3387	M	3.1 V, 142 Hz, 106 µs (mean)	BFMDRS-M (1) 77, (2) 93	NR

Cohen et al. (84)	4	GPi	C + 1−/Med 3387	M	(1) 4.0 V, 130 Hz, 90 µs, (2) 3.4 V, 130 Hz, 120 µs	BFMDRS-M (1) 88, (2) 63 BFMDRS-D (1) 100, (2) 53	(1) 7, (2) 13

Starr et al. (83)^a^	4	PVL-GPi	C + 1−/NR	NR	2.5–3.6 V, 185 Hz, 210 µs (mean)	BFMDRS-M (1) 100, (2) 80, (3) 6, (4) 53	(1) 26, (2) 27, (3) 17, (4) 9

Damier et al. (18); Thobois et al. (9)	3	PVL-GPi	C + 0− or 1−. (Lateral to the AC–PC, anterior to the PC, below the ICL) = (20.1, 15.3, 3.9) (mm, mean)/Med 3387	M	2.5–5.0 V, 130 Hz, 150 µs	ESRS (1) 44, (2) 73, (3) 44, (4) 75, (5) 57, (6) 74, (7) 62 (8) 68, (9) 48, (10) 64 AIMS (1) 50, (2) 62, (3) 35, (4) 58, (5) 37, (6) 67, (7) 33 (8) 78, (9) 69, (10) 67	6

Egidi et al. (85)^a^	4	GPi	NR/Med 3387 and 3389	M	NR, 100–185 Hz, 60–450 µs	BFMDRS-M 47 BFMDRS-D 55 (mean)	NR

Kosel et al. (71)	4	GPi	R C + 4− L C + 1−/Med 3387	M	3.5–3.8 V, 130 Hz, 90 µs	BFMDRS-M 35	18

Magariños-ascone et al. (63)^a^	4	GPi	NR/Med 3389	NR	NR, 60–130 Hz, 90–210 µs	BFMDRS-M 48 BFMDRS-D 44	12

Pretto et al. (72)^a^	4	GPi	NR/NR	NR	4.1 V, 185 Hz, 90 µs	BFMDRS-M 80–90	6

Sako et al. (20)	4	PV-GPi	(1) 3 + 2−, (2), (3), (5) C + 1−, or 2−, (4) R C + 0−1−2−, L C + 1−2−/Med 3387	(1) B, (2)–(5) M	1.6–4.4 V, 60–130 Hz, 450 µs	BFMDRS-M (1) 88, (2) 90, (3) 58, (4) 100, (5) 92, (6) 85 BFMDRS-D (1) 78, (2) 89, (3) 75, (4) 100, (5) 67, (6) 72	(1) 39, (2) 48, (3) 15, (4) 13, (5) 6, (6) 3

Gruber et al. (86)	4	PVL-GPi	(1), (2), (4), (5), (7), (8) 0 + 1− or 1 + 2−, (3), (6) C + 1−/Med 3387 and 3389	(1), (2), (4), (5), (7), (8) B, (3), (6) M	1.4–3.8 V, 130–180 Hz, 60–90 µs	BFMDRS-M (1) 80, (2) 84, (3) 88, (4) 90, (5) 100, (6) 64, (7) 64, (8) 87, (9) 87 BFMDRS-D (1) 95, (2) 50, (3) 77, (4) 67, (5) 100, (6) 25, (7) 33, (8) 63, (9) 100 AIMS (1) 79, (2) 70, (3) 100, (4) 81, (5) 100, (6) 73, (7) 33, (8) 85, (9) 86	(1) 80, (2) 59, (3) 55, (4) 32, (5) 47, (6) 32, (7) 28, (8) 26, (9) 28

Katasakiori et al. (73)^a^	4	GPi	NR/Med 3387	M	NR	BFMDRS-M 94 BFMDRS-D 84	12

Kefalopoulou et al. (62)	4	GPi	C + 0− or 1−/Med 3387	M	2.5–3.6 V, 185 Hz, 250–450 µs	BFMDRS-M 91 AIMS 77	6

Capelle et al. (87)	4	PVL-GPi	1−2 + /Med 3387	B	4.5 V (mean), 130–160 Hz, 90–210 µs	BFMDRS-M (1) 91, (2) 70, (3) 88, (4) 87 BFMDRS-D (1) 88, (2) 50, (3) 100, (4) 50	(1) 27, (2) 30, (3) 16, (4) 36

Chang et al. (88)	4	PV-GPi	C + 1− or 2−. (Lateral to the AC–PC, anterior to the MCP below the ICL) = (20.75, 5.5, 0.65) (mm, mean)/Med 3387	M	2.5–3.6 V, 90–185 Hz, 180–210 µs	BFMDRS-M 71 BFMDRS-D 48 AIMS 77 (mean)	(1) 76, (2) 58, (3) 34, (4) 29, (5) 27

Kim et al. (74)	4	PVL-GPi	NR/Med 3389	M	2.98 V, 89 Hz, 165 µs (mean)	BFMDRS-M 97 BFMDRS-D 100	20

Kovacs et al. (75)	4	PVL-GPi	NR/Med 3389	NR	NR	BFMDRS-M 97 BFMDRS-D 96	12

Spindler et al. (76)	4	GPi	C + 1−/NR	M	3.3 V, 185 Hz, 90 µs	AIMS 67	<60

Woo et al. (89)	4	PV-GPi	C + 1−/Med 3387	M	3.5–3.9 V, 130–180 Hz, 90–210 µs	BFMDRS-M (1) 76, (2) 100, (3) 54	(1) 120, (2) 3, (3) 3

Boulogne et al. (77)	4	PVL-GPi	C + 1−/NR	M	3.5 V, 130 Hz, 90 µs	AIMS 79	120

Trinh et al. (78)	4	GPi	NR/NR	NR	NR	BFMDRS-M 90 BFMDRS-D 87	18

Puri et al. (79)	4	GPi	NR/NR	NR	2.5–3.0 V, 130 Hz, 190 µs	AIMS 55	6

Shaikh et al. (90)	4	GPi	(Lateral to the AC–PC line, anterior to the MCP, below the ICL) = (20.6, 2.9, −1.1) (mm, mean)/NR	M	3.0–4.0 V, 60–185 Hz, 90–450 µs	BFMDRS-M (1) 87, (2) 67, (3) 100, (4) 100, (5) 78, (6) 88, (7) 67, (8) 94	(1) 48, (2) 60, (3) 6, (4) 36, (5) 36, (6) 60, (7) 30, (8) 12

Pouclet-Courtemanche et al. (19)	2 and 3	PV-GPi	Contacts in posteroventral GPi/Med 3387	M	3.17 V, 133 Hz, 120 µs (mean)	ESRS 60 AIMS 63	12 (5 patients) and 72–132 (14 patients)

*NR, not reported; AC, anterior commissure; PC, posterior commissure; MCP, mid-commissural point; ICL, inter-commissural line; Med, medtronic; BFMDRS-M, Burke-Fahn-Marsden Dystonia rating scale motor score, BFMDRS-D, Burke-Fahn-Marsden Dystonia rating scale disability score; AIMS, abnormal involuntary movements scale; ESRS, extrapyramidal symptoms rating scale. Target: PV, posteroventral; PVM, posteroventromedial; PVL, posteroventrolateral; GPi, globus pallidus internus. Mode: B, bipolar; M, monopolar*.

*^a^Reports of patients with tardive syndrome within a larger cohort of dystonia patients*.

### Current Use of GPi-DBS in TDS

Multiple single case reports (62–64, 67–79) and open-labeled small case series (10, 20, 80–90) have shown that GPi-DBS could be highly effective in the treatment of patients with medically intractable TDS (see Tables 2 and 3). Recently, a class II study provided level C evidence for positive effects of GPi-DBS in TDS therapy (19). Here, we introduce the current state of GPi-DBS use in the treatment of patients with TDS.

#### Patient Selection

Selection of candidates for GPi-DBS is a critical step for obtaining good outcome results and for avoiding adverse events. The primary inclusion criterion is that patients experience medically intractable and markedly disabling motor symptoms associated with TDS. According to the criteria proposed by The French Stimulation for TDD (STARDYS), which might so far be the most rigorous and strict, DBS should only be considered for patients with persistent (>1 year) and severely disabling TDS, for whom treatment with clozapine or tetrabenazine at their maximum tolerable dosages had been attempted for at least 4 weeks (18, 19). The exclusion criteria are essentially the same as those applied to patients with primary dystonias, which include marked cognitive impairment, acute psychiatric changes, severe depression, and other coexisting medical disorders that would increase the surgical risk (86, 91). To predict the potential risks inherent to the surgical procedures, a preoperative brain MRI should be performed to check for the presence of brain atrophy and/or other organic lesions. It is also important to evaluate if the psychiatric conditions of the patient are satisfactorily stable with the current medication, for at least several months prior to the surgery and to confirm the ability to provide consent for the surgical procedure (18, 19).

#### GPi Target Determination

**Figure 1:**
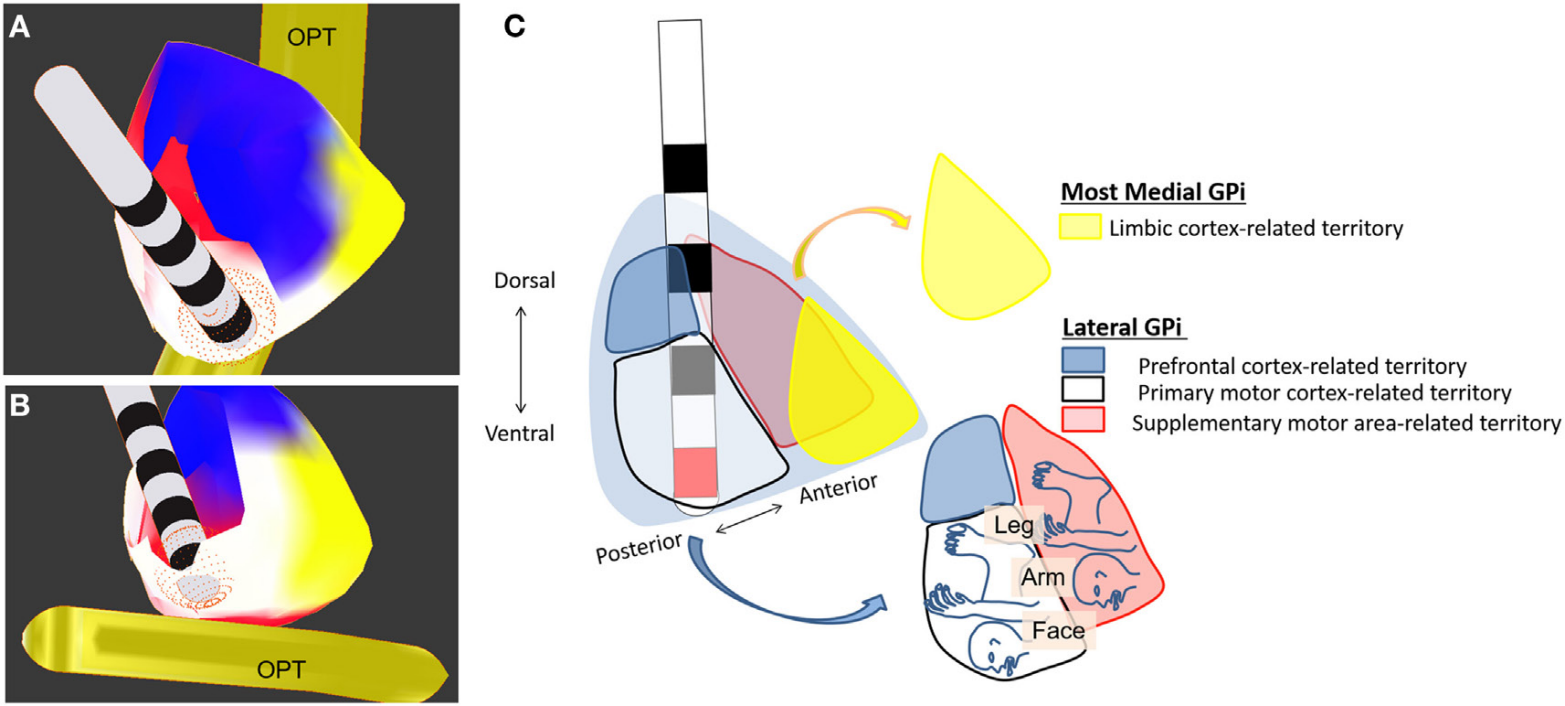
**Deep brain stimulation of the globus pallidus internus (GPi)**. Electrodes are placed in the ventroposterolateral part of the GPi (the posterodorsolateral part of the GPi is partially removed). **(A)** Dorsoposterior view of the GPi. **(B)** Ventroposterior view of the GPi. **(C)** Schematic drawing of GPi-DBS with active contact (red) placed within the posteroventrolateral GPi. Colors indicate the territories receiving limbic- (yellow), prefrontal- (blue), motor- (white), and supplementary motor (red) cortex-related inputs. OPT, optic tract.

So far, bilateral DBS targeting the posteroventral part of the GPi has been used in patients with TDS (see Table 3). More specifically, the posteroventrolateral part of the GPi was chosen as the optimal target in most previous reports (9, 10, 18, 74, 75, 77, 83, 86, 87) but the posteroventromedial part of the GPi was also targeted in two reports (67, 81) (Figures 1A–C). Ventral two-thirds of the posterior GPi is the primary motor cortex-related territory that shows a somatotopic organization (Figure 1C) (92). The supplementary motor area-related territory locates more dorsal and anterior to the motor cortex-related territory (92). Dorsal one-third of the posterior GPi is the prefrontal cortex-related territory, while the most medial part of the GPi corresponds to the limbic cortex-related territory (92). Imaging with stereotactic MRI or CT-MRI fusion method is usually employed to define the anatomical targets (76). The stereotactic coordinates for the GPi are 19–22 mm lateral to the anterior commissure–posterior commissure line, 2–4 mm anterior to the mid-commissural point, and 4–6 mm inferior to the intercommissural line (10, 62, 63, 70, 73, 74, 80–82, 84, 86, 87, 89). Pouclet-Courtemanche et al. suggested that the locations of active electrodes as far as they were positioned within the posterolateral part of the ventral GPi might not be optimal in terms of clinical benefit (19). In a previous case report, a target that was 1–2 mm above, 1.5 mm rostral, and 2 mm medial to the usual target in dystonia was chosen to selectively stimulate the facial area (71). However, this single case was an exception because, as shown in Table 3, the GPi active contacts that are usually used are the same as in primary dystonia. During surgery, MERs are often used to detect neuronal discharges in the GPi. Intraoperative macrostimulation has also been used to assess the therapeutic effects of DBS and to determine thresholds for capsular stimulation and visual phosphene detection (76).

#### Stimulating Paradigms

Postoperatively, most ventral contacts of the DBS leads located within the GPi were usually used with the monopolar stimulating modes (9, 10, 16, 18–20, 62, 67, 69–71, 74, 76, 77, 80–82, 84–86, 88–90), and rarely with the bipolar modes (20, 64, 68, 86, 87) (Table 3). Stimuli were applied with amplitudes ranging from 2.7 to 4.5 V and a high frequency setting (>100 Hz) with a pulse width of 60–240 µs; alternatively, low frequency stimulation (<100 Hz) with a pulse width of 120–450 µs was also often applied (10, 18–20, 67, 68, 71, 74, 76, 77, 79, 84–90) (Table 3). The stimulation parameters used in TDS were similar to those applied in primary dystonia.

#### Effects on Motor Symptoms

Data from the STARDYS study group (18, 19) have verified the beneficial effects of bilateral GPi-DBS in patients with TDS. Following a prospective multicenter trial using double-blind evaluations at 6 months after surgery, reports showed that in all patients, the extrapyramidal symptoms rating scale (ESRS) scores decreased to less than 60% of the preoperative baseline, and that there was a 49% reduction of the total ESRS scores in the stimulation “on” conditions when compared to the “off” conditions. Pouclet-Coutemanche et al. showed that this therapeutic impact remained at 12 months after surgery, with a 58% (*p* < 0.0001) decrease of the total ESRS scores and a 50% (*p* < 0.0001) decrease of the total AIMS scores (19). Given the results obtained from the study with long-term (6–11 years) follow-up with the patients (*n* = 14), they also reported a persistent improvement of TDS patients’ conditions, with a 60 and 63% decrease from preoperative baselines in the total ESRS and AIMS scores, respectively (19).

Multiple case reports document that TDS-associated motor symptoms could be alleviated immediately or within a few days after the GPi-DBS was initiated (10, 67, 75, 77, 81, 82, 84, 86, 87). Among the TDS symptoms, choreiform dyskinesia tended to respond to DBS earlier than tonic postural dystonia, which gradually improved over weeks or months (18, 68, 70, 77, 84, 87, 88). Therapeutic efficacy of GPi-DBS seemed to be higher in the choreiform and dystonic movements than in the fixed dystonias (10, 20, 86, 90). Shaikh et al. reported that meaningful improvements in neck and truncal dystonias were most challenging to achieve, but could develop gradually over 48 months after the stimulation was initiated (90). Prospective studies with blind assessments also showed that GPi-DBS could alleviate TDS symptoms regardless of their subtypes (e.g., chorea and dystonia) or body distributions (18, 19).

The beneficial effects from GPi-DBS could produce an improvement in daily life activities in patients with disabilities due to TDS. Using the Burke-Fahn-Marsden Dystonia rating scale (BFMDRS), a systematic review showed that GPi-DBS produced a 74% improvement of disability scores (*p* < 0.0001) (93). Using the 36-item Short Form General Health Survey, Gruber et al. also reported a 46% improvement in total subscores for physical health (86). However, a prospective study using Lehman quality of life (QOL) Interview showed no significant change in QOL before and 6 months after surgery in seven patients with TDS (19).

#### Effects on Non-Motor Symptoms

Two separate case series reports (71, 86) show that GPi-DBS produced a significant improvement of mood in patients with TDS, as determined by the Hamilton rating scale for depression, the Beck Depression Inventory Score, and the Montgomery-Åsberg Depression rating scale (MADRS). However, a prospective study on 16–19 patients found that the mean scores of both the MADRS and the positive and negative syndrome scale did not change significantly up to 1 year after surgery (19).

Gruber et al. reported no significant change in cognitive functions of nine patients before and after surgery, as determined by the Mattis Dementia rating scale (MDRS), the Multiple Wording Test part B, the Rey Auditory Verbal Learning Test, and the digit span task (86). Pouclet-Courtemanche et al. published a prospective study on 16–19 patients to show the results of neuropsychological tests using Mini-Mental State Examination (MMSE), the Frontal Assessment Battery (FAB), and the MDRS (19). They found that there were no significant changes in the mean scores of both the MMSE and FAB up to 1 year after surgery, while the mean scores of the MDRS improved at 3 months and persisted for 1 year after surgery (*p* < 0.05). Thus, it is likely that in TDS patients, GPi-DBS might not exert a negative impact on QOL, mood, or cognition.

#### Adverse Events

The overall complication rate of GPi-DBS for TDS is 9%, which is almost equivalent to that of GPi-DBS for other movement disorders (76, 94). There are no reports of death related to DBS in patients with TDS (95). However, a potential risk of suicide after GPi-DBS surgery has been suggested in patients with TDS (76, 96). Complications relating to the implanted DBS devices in patients with TDS, such as displacement and misplacement of the DBS leads, have also been noted (19, 87). Pouclet-Courtemanche et al. reported other complications that include dysfunction of the active contacts, painful traction by the cable connection, and sudden stopping of the stimulator (19). Surgery-related infection (80, 88) and venous infarction (83, 88) have also been documented. Electrostimulation-dependent complications, such as paresthesia, shuffling gait, decreased sensitivity for precise and skillful movements, muscular contractions, phosphenes, scotoma, and dysarthria, have also been reported (67, 69, 80, 82, 87), although they were transient and addressed by adjustment of settings. Concerning psychiatric issues, Trottenberg et al. reported that one of five patients with schizophrenia manifested a psychotic relapse 6 months after surgery (82). Pouclet-Courtemanche et al. reported that within 1 year after surgery, 8 of 19 patients experienced adverse psychiatric events that included depression, anxiety, manic states, delirium, agitation, and aggressiveness, although mental health was successfully restored with medical treatments (19).

#### Could STN-DBS Be a New Target for TDS?

Two separate case series reports document that STN-DBS produced striking improvement of motor symptoms in patients with TDS, as determined by the BFMDRS (65, 66). The average score improved by 89% compared to the baseline. Sun et al. reported that STN-DBS produced immediate symptomatic improvement, using lower stimulation parameters with longer battery life (66). They proposed that STN-DBS might enable better symptomatic control over GPi-DBS. However, there is currently a lack of head-to-head comparison between GPi and STN stimulation for primary dystonia and TDS (97). Furthermore, the effects of STN-DBS on the non-motor symptoms of TDS patients are still unknown. Several recent meta-analyses comparing the effects of GPi-DBS with STN-DBS in patients with Parkinson’s disease concluded that the risk of worsening depression with GPi-stimulated patients was the same or even smaller than that with STN-stimulated patients (98–101). A selective decline in cognitive functions with STN-DBS has also been highlighted in almost all the meta-analyses (98–103). These observations could be in part attributed to the reduction in dopaminergic drugs for STN-stimulated patients (99, 100, 104). Given the extent of dopamine withdrawal in STN-stimulated patients in Parkinson’s disease, the results of studies comparing GPi and STN stimulations in these patients cannot be directly applied to primary dystonia or TDS. Therefore, well-designed randomized controlled trials will be required to select better targets for patients with dystonia, including those with TDS.

## Summary

Globus pallidus internus-DBS results in promising and continuous improvement in motor function over months and possibly years, which may persist over 6–11 years in patients with TDS. There is no available evidence to demonstrate that GPi-DBS negatively impacts QOL, mood, or cognition in patients with TDS. The complication rate of GPi-DBS for TDS is almost equivalent to that of GPi-DBS for other movement disorders. To obtain a higher level of clinical evidence about the precise efficacy of GPi-DBS in reducing TDS, more well-designed double-blind trials are needed. In particular, it is important to clarify specific inclusion criteria for patient selection. One of the particular questions to be addressed in the near future is a comparison of STN-DBS and GPi-DBS efficacy in patients with TDS.

## Author Contributions

The conception or design of the work: RM and SG. The acquisition, analysis, or interpretation of data for the work: RM and HM. Drafting the work: RM and SG. Revising the work critically for important intellectual content: SN, RK, and SG. Final approval of the version to be published and agreement to be accountable for all aspects of the work in ensuring that questions related to the accuracy or integrity of any part of the work are appropriately investigated and resolved: RM, HM, SN, RK, and SG.

## Conflict of Interest Statement

The authors declare that the research was conducted in the absence of any commercial or financial relationships that could be construed as a potential conflict of interest. The reviewers H-KM and handling Editor declared their shared affiliation, and the handling Editor states that the process nevertheless met the standards of a fair and objective review.
